# Over-the-Scope Clip for Bleeding Malignant Gastric Ulcer

**DOI:** 10.14309/crj.0000000000000611

**Published:** 2021-07-20

**Authors:** Diego Colom Steele, Tarun Rustagi

**Affiliations:** 1 Division of Gastroenterology and Hepatology, Department of Internal Medicine, University of New Mexico School of Medicine, Albuquerque, NM

A 78-year-old man presented with recent onset hematemesis, melena, and abdominal pain associated with acute anemia (hemoglobin 5.6 g/dL and decreased from 16.2 g/dL). Esophagogastroduodenoscopy revealed a malignant-appearing large (5 cm) deeply cratered ulcer with heaped-up margins involving the antrum. A large (5 mm) protuberant pulsatile visible vessel was noted within the ulcer base with adjacent blood clots (Figures 1 and 2). A 11/6-gc over-the-scope clip (OTSC; Ovesco Endoscopy AG, Tubingen, Germany) was deployed over the visible vessel. The clip was positioned with vessel in the center of the clip with adequate compression (Figures 3 and 4). No bleeding was noted during the endoscopy. Biopsies of the ulcer margins showed a poorly differentiated invasive adenocarcinoma. There was no clinical evidence of recurrent bleeding, and he was discharged 2 days later.

**Figure 1.**
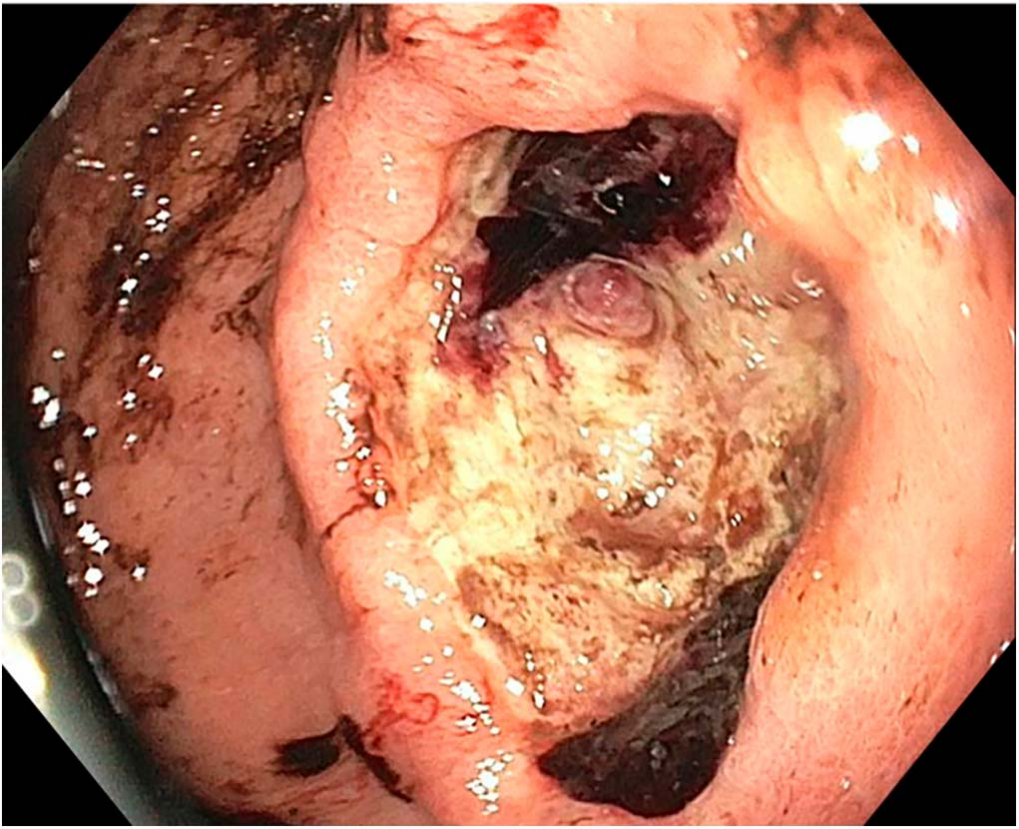
Large malignant-appearing antral ulcer with a visible vessel and adjacent blood clot.

**Figure 2.**
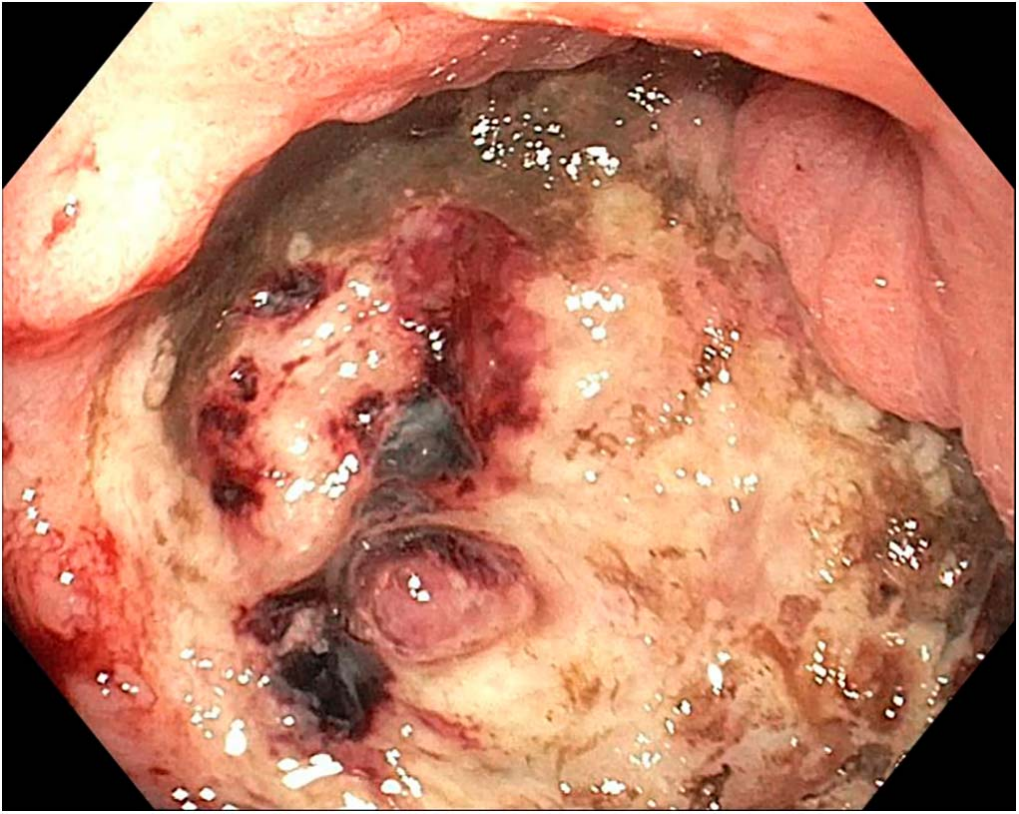
Large protuberant visible vessel within the ulcer base.

**Figure 3.**
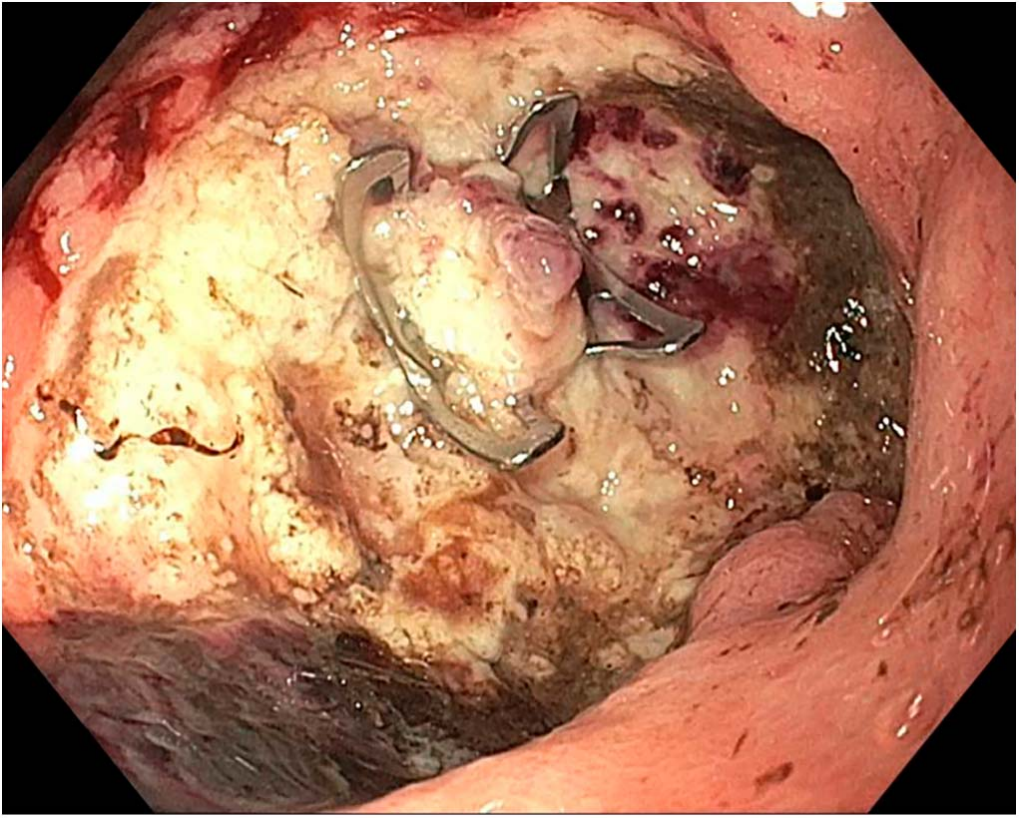
Over-the-scope clip was placed over the vessel in the ulcer base.

**Figure 4.**
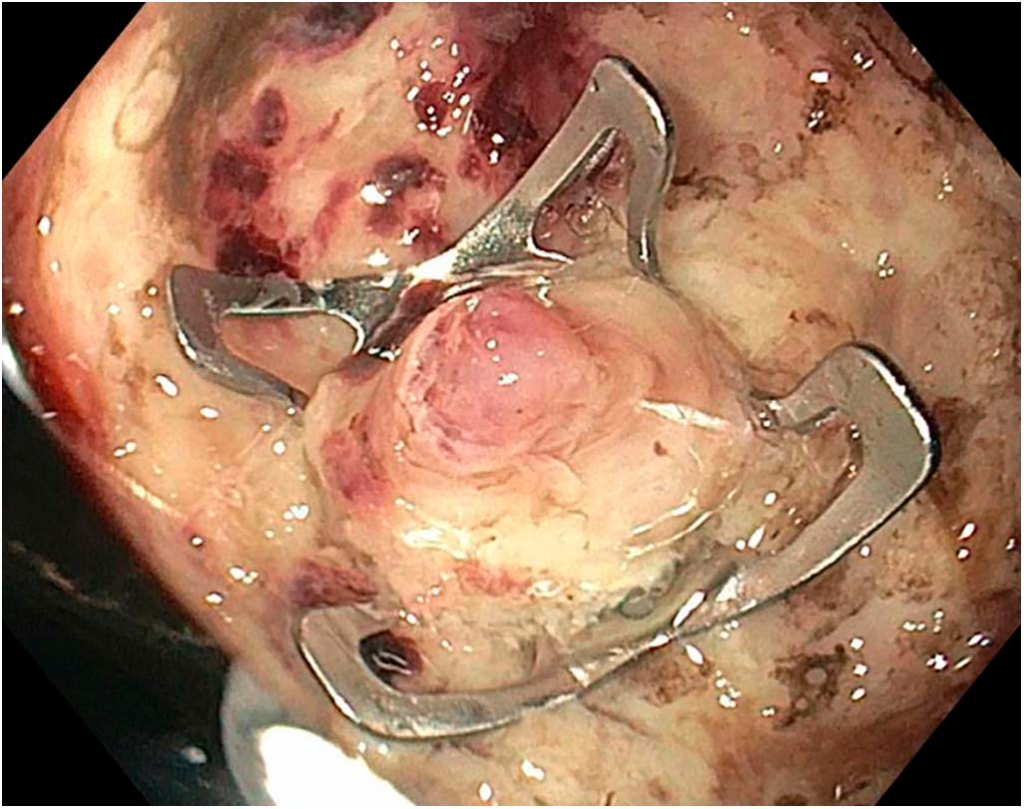
Over-the-scope clip was positioned with large vessel in the center of the clip.

OTSC technology has been available for several years, but there are little data on its efficacy and outcomes in malignant upper gastrointestinal bleeding. In this case, the visible vessel was large and located within a fibrotic base making use of conventional means such as through-the-scope hemostasis clips or contact thermal therapy difficult with increased risk of intraprocedural and recurrent bleeding; therefore, OTSC was successfully used with adequate hemostasis without complications.

## DISCLOSURES

Author contributions: DC Steele wrote the article. T. Rustagi edited the article, revised the article for intellectual content, and is the article guarantor.

Financial disclosure: None to report.

Informed consent was obtained for this case report.

